# When ASFV Infection Meets the cGAS-STING Signaling Pathway

**DOI:** 10.1155/2024/6898157

**Published:** 2024-04-08

**Authors:** Songbiao Chen, Ke Shang, Ying Wei, Jian Chen, Zuhua Yu, Lei He, Ke Ding

**Affiliations:** ^1^Laboratory of Functional Microbiology and Animal Health, College of Animal Science and Technology, Henan University of Science and Technology, Luoyang 471003, China; ^2^Luoyang Key Laboratory of Live Carrier Biomaterial and Animal Disease Prevention and Control, Henan University of Science and Technology, Luoyang 471003, China; ^3^The Key Lab of Animal Disease and Public Health, Henan University of Science and Technology, Luoyang 471023, China; ^4^Ministry of Education Key Laboratory for Animal Pathogens and Biosafety, Zhengzhou 450000, China

## Abstract

The African swine fever virus (ASFV) has the ability to infect both wild boars and domestic pigs, regardless of their breeds or ages, often resulting in a mortality rate of 100%. Host innate immunity is the most important defense weapon against invasion of pathogenic microbial infection. cGAS-STING signaling pathway is one of the greatest discoveries of the twenty-first century, which is crucial in host's innate immune response. Recent studies have found that the interaction between cGAS/STING pathway and ASFV plays a key role during ASFV infection. At the same time, ASFV has also evolved different strategies to evade the killing of the host cGAS/STING pathway and promote its survival. Here, we review the latest progress in the interaction between ASFV infection, cGAS/STING pathways, and their related molecular mechanisms, aiming to provide new ideas for further research on the pathogenesis of ASFV, the development of vaccines and therapeutic drugs.

## 1. Introduction

African swine fever virus (ASFV) causes a devastating disease in pigs [1, 2], it was the first outbreak in Kenya and was reported in 1921 [3]. Subsequently, ASFV was found in China on August 3, 2018 [4, 5], and soon spread across the country with unprecedented speed, which caused at least 300 million pigs estimated to have died from infection or preventive culling, with economic losses of about $100 billion [6].

ASFV infection is the product of the interaction between virus and host antiviral [7–9]. Innate immunity, also known as natural immunity or nonspecific immunity, is the most important host defense weapon against pathogenic microbial invasion [10, 11]. The immune system depends on a group of receptors known as pattern recognition receptors (PRRs) to detect pathogen-associated molecular patterns (PAMPs) and trigger the production of immune molecules and cytokines in host cells to fight pathogen microbial invasion [12]. DNA is one of the important PAMPs, once the pathogen enters the host cell, it will release its DNA into the cytoplasm. The DNA sensor cGAS is able to detect cytoplasmic DNA and promptly activate the innate immune response through the STING protein, which is mediated by interferon-stimulating factor [13, 14]. Following that, it was discovered that STING plays a role in transmitting signals for type I interferon (IFN-I) through interferon regulatory factor 3 (IRF3) [15, 16], and it was also found that STING dimerization is crucial for initiating subsequent signals [17, 18].

cGAS-STING is crucial for anti-DNA virus and RNA virus infection [19–22]. Recent studies have confirmed that cGAS-STING is essential for ASFV infection and pathogenesis. Meanwhile, ASFV has also developed a series of strategies to manipulate the host's defense function against viral infection [23–25]. Hence, thorough investigation into ASF immune evasion theory is crucial, alongside enhancing biosafety measures to combat the ASF outbreak and expediting the creation of reliable vaccines. At present, the understanding of the pathogenesis of ASFV is incomplete, which seriously affects the effective prevention and development the efficient vaccines. Here, we review the interaction between ASFV and cGAS-STING and their related molecular mechanisms, aiming to provide new ideas for further research on the pathogenesis of ASFV and effective vaccines.

## 2. Overview of ASFV

**Figure 1:**
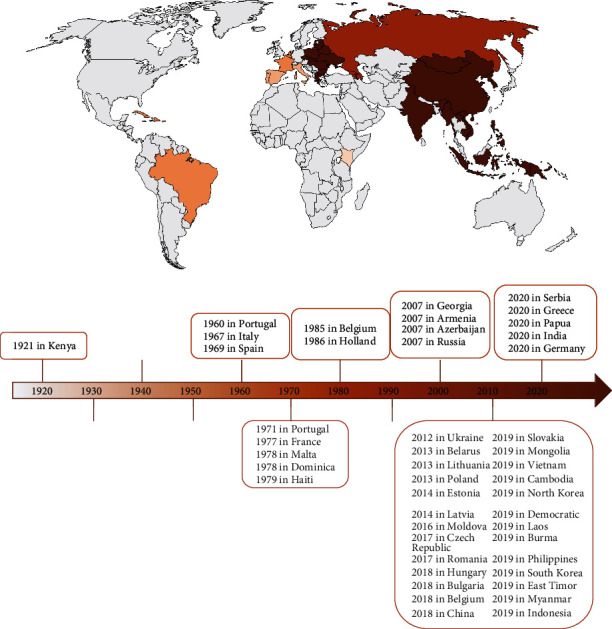
The current global epidemiological scenario for ASFV (updated to 2020). Note. The lighter the color, the earlier the first outbreak.

**Figure 2:**
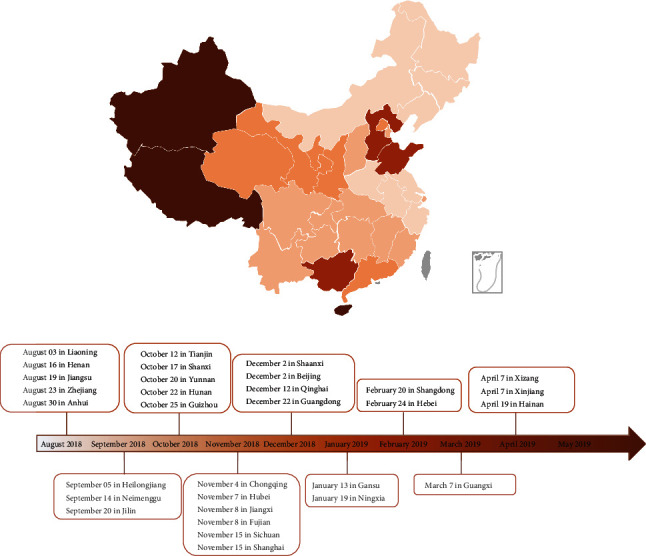
The current China epidemiological scenario for ASFV outbreak between August 3, 2018 and April 19, 2019. Note. The lighter the color, the earlier the first outbreak.

ASF has spread to more than 40 countries all over the world since its discovery in 1921 [3] (Figure 1, incomplete statistics until 2020). The first indigenous case of ASF was first reported in Liaoning, China, on August 3, 2018 [4, 5], and soon spread with unprecedented speed to all 30 provinces and autonomous regions of the country within 8 months (Figure 2, updated to April 19, 2019). Vaccination is the most effective approach to prevent and control infectious diseases [26, 27]. At present, there is still a lack of safe and effective ASF vaccines, so vaccine development remains a global challenge [28]. The most prominent feature of ASFV is its ability to camouflage to evade the host immune system and establish a complete infection, which is also the key factor hindering ASF prevention, control, and development [29].

Currently available data have shown that ASFV can replication in primary cells and cell lines [30–32]. Among them, ASFV had the best proliferation in alveolar macrophages [33–35]. During infection, ASFV initially replicates in monocytes and macrophages located in the tonsil and mandibular lymph nodes, before spreading to other tissues through the bloodstream and/or lymphatic system [35]. Among, the spleen is the organ with the highest viral load and the most serious pathological manifestations in all organs of pigs. Infection with ASFV leads to the demise of numerous monocytes in the spleen, leading to suppression of interferon response and antigen expression, ultimately facilitating the prolonged infection of ASFV [36, 37]. In the initial stage of ASFV replication, virions first interact with cell surface receptors, attach to cells, and then enter into macrophages mainly through clathrin-mediated endocytosis [38, 39] and micropinocytosis [40]. Prior research has indicated that host proteins CD1d [41], CD163 [42–45], and SIGLEC1 [42] may facilitate ASFV entry into cells; however, the role of CD1a, CD163, and SIGLEC1 as ASFV receptors requires additional confirmation. After the virion enters the cell, it travels from the early endosome to the late endosome in a pH-dependent manner along the endolysosome pathway and uncoating in this place. The virus particles expose the inner envelope, allowing it to interact, and then fuse the viral membrane with the restrictive membrane of the endosome. The bare kernel can be released into the cell solutes and transported to the perinuclear viral replication factory to begin replication [46, 47].

## 3. Overview of cGAS-STING Signaling Pathway

**Figure 3:**
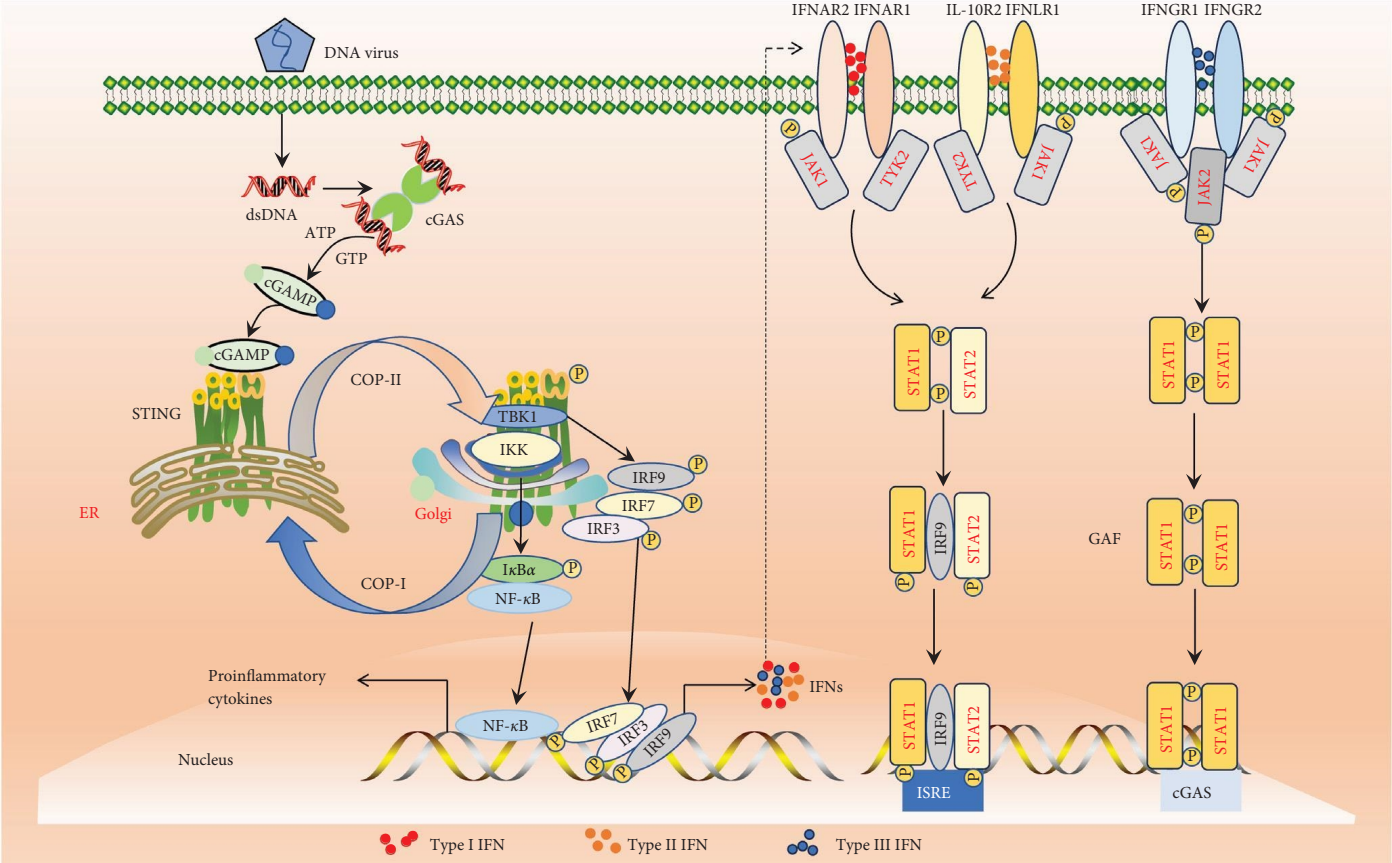
Schematic diagram of cGAS/STING signal pathway.

cGAS was first discovered in mammalian cells in 2013 and can synthesize cGAMP as a second messenger to directly activate STING [13–48]. Following infection of host cells by pathogenic microorganisms, cGAS as a crucial DNA sensor, quickly identifies and detects foreign viral DNA in the cytoplasm, leading to the formation of a 2 : 2 dimer and the generation of 2′,3′-cGAMP. Subsequently, 2′,3′-cGAMP binds to STING on the ER membrane, triggering activation through a change in structure. Activated STING then moves from the ER to the Golgi intermediate compartment. Upon activation, STING recruits active TBK1 using its carboxyl-terminal, forming the STING-TBK1 complex on the Golgi membrane. This complex further triggers IRF3 phosphorylation, leading to IRF3 dimerization in the nucleus (Figure 3). Simultaneously, STING has the ability to directly trigger the I*κκ* kinase, leading to the phosphorylation of the inhibitor of NF-*κ*B, ultimately resulting in the degradation of the phosphorylated I*κ*B through the ubiquitin-proteasome pathway. NF-*κ*B enters the nucleus and phosphorylates IRF3 to induce the secretion of interferon and inflammatory cytokines to promote host anti-infective immunity [49, 50].

### 3.1. The cGAS-STING Signaling Pathway and Interferon

IFNs are the initial defense mechanism of the host against viral infections, with three distinct categories: IFN-I, IFN-II, and IFN-III [51, 52]. Activation of cGAS/STING can stimulate the production of IFNs, which then trigger the transcription of numerous downstream interferon-stimulating genes by interacting with various IFN receptors located on the cell membrane [53, 54]. IFN-I functions by attaching to IFN-*α* receptor IFNAR1 and IFNAR2, while IFN-III binds to IL-10R2 and IFNLR1. IFN-II forms a dimer through a heterodimer consisting of IFN-*γ* receptors IFNGR1 and IFNGR2 [55] (Figure 3). When IFN-I and IFN-III bind to receptors, it triggers the activation of janus kinase 1 (JAK1) and tyrosine kinase 2 (TYK2), resulting in the phosphorylation of signal transduction and transcriptional activators 1 and 2 (STAT1 and 2). STAT1/STAT2 combine to create a heterodimer, which then brings in IFN regulatory factor 9 (IRF9) to produce IFN-stimulating gene factor 3 (ISGF3) [56, 57] (Figure 3). When IFN-II binds to the receptor, it causes JAK1 and JAK2 tyrosine kinases to be phosphorylated, resulting in the phosphorylation of STAT1. The phosphorylated STAT1 then forms an IFN-*γ* activator (GAF) dimer, which activates the expression of antiviral genes [56] (Figure 3).

### 3.2. The cGAS-STING Signaling Pathway and Cell Death

cGAS-STING is involved not just in the release of IFN and inflammatory cytokines, but also plays a role in various cell death mechanisms including apoptosis, necroptosis, pyroptosis, and ferroptosis [58–60].

**Figure 4:**
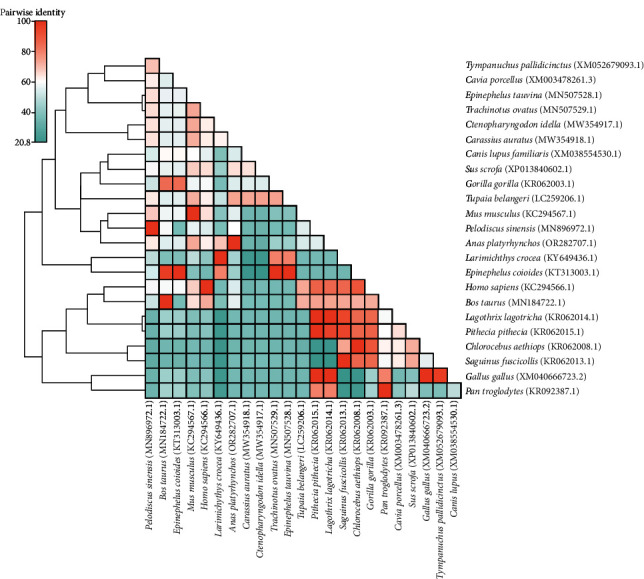
Two-dimensional pairwise identity plot of cGAS amino acid sequences of different species constructed using BioAider V1.314 (https://www.chiplot.online/#Radar-plot). The grids of different colors indicate different identities, which were analyzed via clustering.

Activation of the cGAS-STING pathway may trigger cell apoptosis [61, 62]. Stimulation with IFN-*β* can trigger apoptosis in cells by activating JAK-STAT, inhibiting the PI3K/AKT pathway, leading to the release of cytochrome C in the cytoplasm, and subsequently activating caspase-9 through cytochrome C [63]. Meanwhile, IFN-*β* can also induce apoptosis dependent on caspase-8 [64], and downregulation of IRF3 can negative regulation the proapoptotic effect of IFN-I [65–67]. The levels of cGAS, STING, and NLRP3 were significantly upregulated in intervertebral disc degeneration (IDD) patients, and epigallocatechin gallate (EGCG) treatment could inhibit cell apoptosis by target cGAS/STING/NLRP3 [68, 69] (Figure 4).

The cGAS-STING signaling pathway promotes potassium efflux activation of NLRP3 and induces pyroptosis by regulating STING transport [70], although the molecular mechanism is still unknown (Figure 4). Furthermore, cGAS-STING is capable of stimulating the production of mixed kinase domain-like protein (MLKL), a crucial element in necroptosis [71]. Additionally, proapoptotic p53 can boost the phosphorylation of receptor-interacting serine/threonine kinase 3 (RIPK3) and MLKL, leading to the release of mitochondrial DNA (mtDNA) and activation of STING, ultimately resulting in necroptosis [72] (Figure 4). Ferroptosis is identified by lipid peroxidation that relies on iron, and it is triggered by STING activation due to lipid peroxidation instead of IFN signaling when faced with mtDNA stress [73, 74] (Figure 4).

### 3.3. The cGAS-STING Signaling Pathway and Mitochondrial Dysfunction

Mitochondria are the energy factories of living organisms [75, 76]. Additionally, mitochondria play a key role in producing reactive oxygen species (ROS) in living organisms, particularly within the electron transport system (ETS) [77]. Under normal physiological conditions, these ROS may be secondary messengers that control the intracellular signal transduction cascade [78–80]. However, large amounts of ROS production can lead to oxidative stress [75].

**Figure 5:**
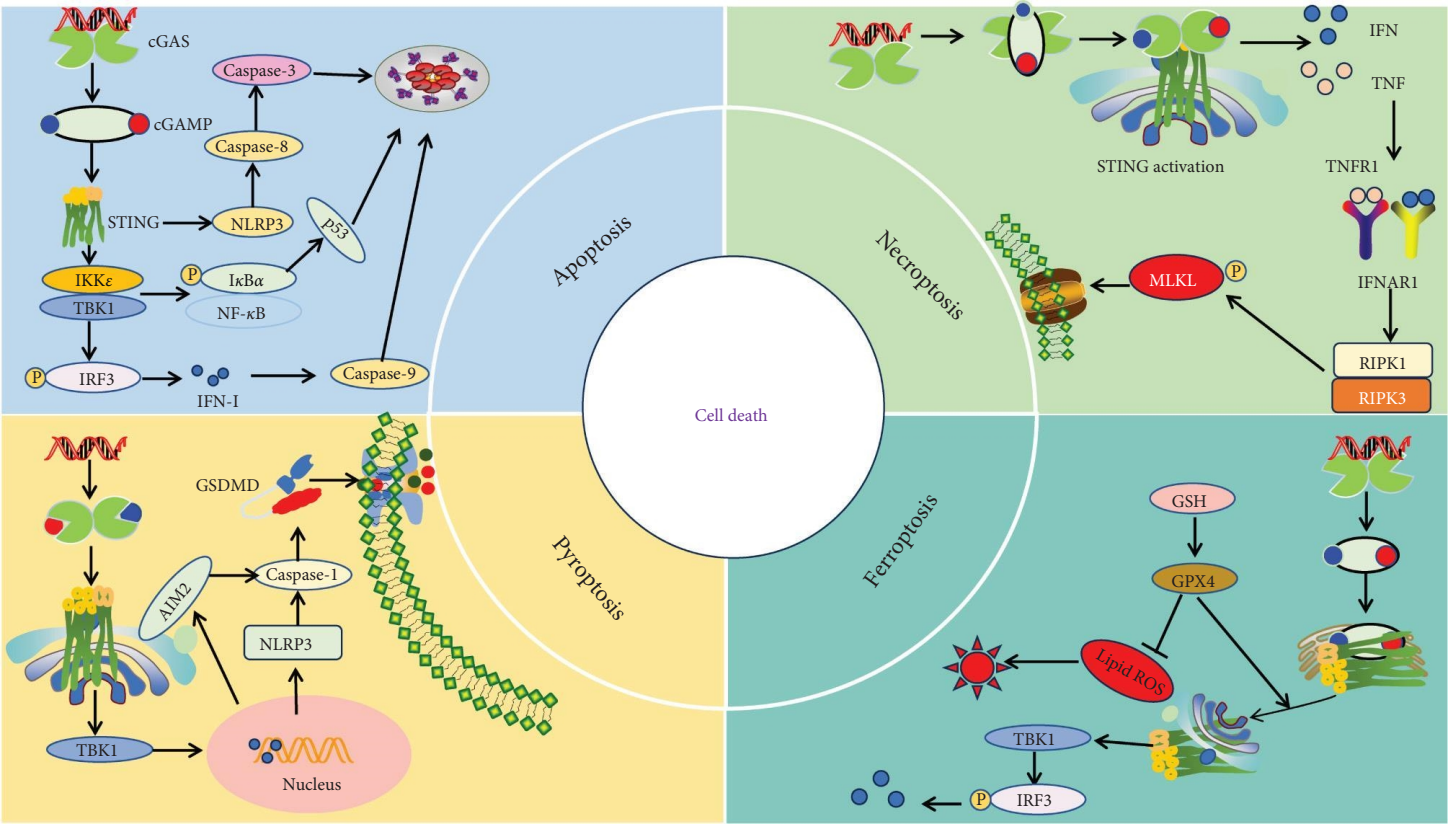
The crosstalk between cGAS-STING signaling pathway and cell death.

ROS production and Ca^2+^ ion accumulation can trigger the activation of mitochondrial permeability transition pore (mPTP), resulting in the release of proapoptotic molecules like cytochrome C, ultimately leading to apoptosis or necroptosis (Figure 5). Apoptosis leads to the creation of large pores in the mitochondrial outer membrane (MOM) by BAX and BAK [81, 82], allowing mtDNA to move from the pore to the cytoplasm via the cGAS-STING pathway, activating the innate immune response [83].

## 4. Interaction between cGAS-STING Signaling Pathway and African Swine Fever Virus

Viral infection depends on the interaction between virus and host proteins. The ASFV p72 protein is a key element of the ASFV capsid, which is essential for virus attachment and entry [84–86]. Recent research indicates that p72 has the ability to bind with CD63, B2M, YTHDF2, etc., cellular proteins. The bioinformatics analysis of Go enrichment and protein interaction network revealed that the host proteins played important roles in virus attachment, invasion, replication, assembly, and immune regulation [87]. Furthermore, Sun et al. [88] also screened that p72 can interact with 2′,5′-oligoadenylate synthetase gene 1 (OAS1), which is a interferon-stimulating gene mediated by cGAS-STING activation. After their interaction, TRIM21 is recruited through the ubiquitin-proteasome pathway to degrade p72 at the K63 site, further destroying the assembly of mature ASFV particles and reducing the production of mature virions [88]. Wu et al. [89] found that the protein–protein interaction (PPI) network between ASFV and host immune pathways revealed that certain genes such as A151R, MGF360-11 L, E165R, G1340L, MGF505-3R, and A137R have a notable suppressive impact on IFN-*β* production. Additionally, K421R, MGF360-11L, EP364R, C147L, A151R, and A137R were shown to significantly inhibit NF-*κ*B production [89]. IFN-*β* and NF-*κ*B are identified as downstream components of the cGAS-STING pathway, indicating that ASFV might manipulate the host cGAS-STING pathway by targeting these proteins to suppress the host immune response and enhance viral replication.

ASFV can target cGAS-STING [90], NF-*κ*B [91], TGF-*β* [92], ubiquitination [93], and apoptosis [94] signaling pathway promotes viral replication. The cGAS-STING pathway is crucial in defending against DNA viruses in the host and is the most extensively researched ASFV pathway in terms of host pathogenicity and immune evasion. cGAS-STING has strict and fine regulations in space and time to ensure that while eliminating pathogen infection, it avoids damage to the body due to insufficient or excessive immune response [95].

Replication strategies of ASFV strains and cell tropism vary widely [25]. Over the long course of evolution, ASFV encodes and expresses multiple immune escape proteins to antagonize the host immune response. The escape of ASFV to the natural immune system of the host is an important strategy for causing persistent infection of the virus, and it is also an important reason for the low immune protection and persistent infection during the development of live attenuated vaccine [96]. Earlier research has shown that the ASFV genetic material is capable of producing various viral proteins that disrupt the production of host cell proteins and disrupt pathways, ultimately hindering and avoiding the host's immune response while promoting its own growth and spread [97, 98] (Table 1).

Below, we will explain how ASFV hinders IFN-I and enhances viral replication by interacting with signal molecules on various cGAS-STING signaling pathways at the molecular level.

### 4.1. Targeting cGAS Inhibits the Molecular Mechanism of cGAS-STING Activation

**Figure 6:**
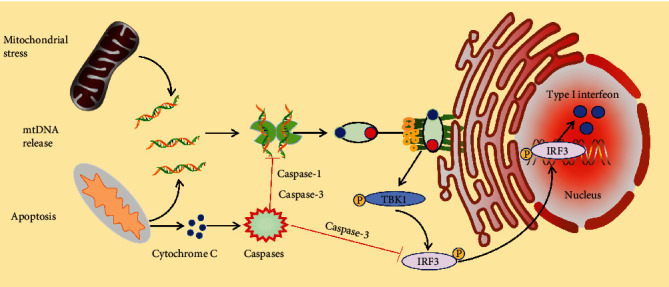
cGAS-STING signaling pathway can impact mitochondrial function and contribute to mitochondrial dysfunction, leading to a feedback loop between mitochondrial metabolism and innate immunity.

cGAS, also known as C6ORF150 or MB21D1, is a member of the cGAS/DNCV-like nucleotide transferase (CD-NTase) superfamily. The cGAS gene is well-preserved across human, mouse, pig, and chicken species (Figure 6). The catalytic domain is a two-leaf structure composed of overlapping nucleotide transferase core domain (NTase) and Mab21 domain. Including a central catalytic domain and two different positively charged surfaces [125, 126]. It functions as a DNA receptor in mammals, can recognize cytoplasmic DNA and produce cGAMP, and activate STING regulate the secretion of downstream IFN-I and other cytokines.

**Figure 7:**
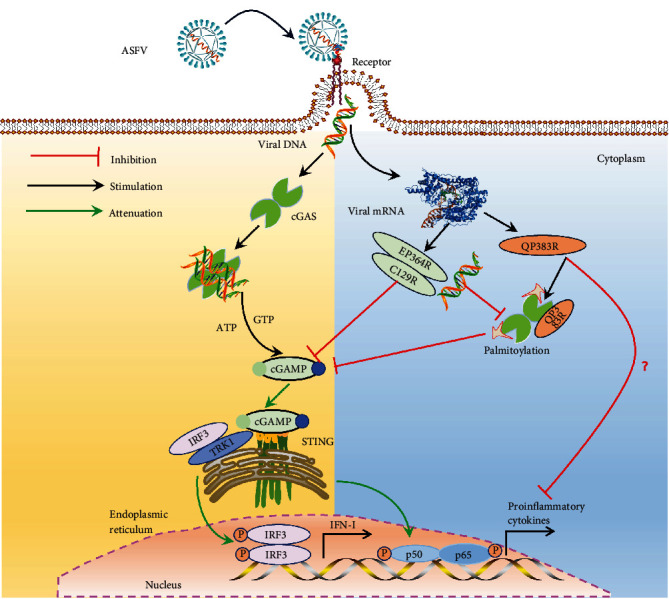
ASFV proteins modulating cGAS and cGAMP inhibit the molecular mechanism of cGAS-STING signaling pathway activation.

QP383R of ASFV is an unnamed protein composed of 383 amino acids. QP383R inhibits the inflammatory response during ASFV infection by inhibiting AIM2 inflammasome activation [127] (Figure 7). Additionally, QP383R functions as a suppressor of cGAS/STING-driven innate immune responses, with elevated levels of QP383R leading to reduced activation of cGAS-STING by dsDNA [99] (Figure 7). Further studies showed that QP383R promoted the palmitoylation of cGAS through its C-terminal (284-383 aa) interaction with the nucleotide transferase (NTase) domain of cGAS, inhibited the binding of cGAS to ligand DNA and cGAS dimerization reduced the downstream interferon reaction [99] (Figure 7).

### 4.2. Targeting 2′,3′-cGAMP Inhibits the Molecular Mechanism of cGAS-STING Activation

Wu et al. [14] discovered cGAMP as a natural signaling molecule for innate immunity in response to cytoplasmic DNA in 2012 and confirmed its presence in mammalian cells. Subsequently, it was confirmed that cGAMP is the catalytic product of cGAS (the substrate is ATP and GTP) by liquid chromatography–mass spectrometry, and the small molecule cGAMP directly binds and activates STING to induce IFN-I production [13].

After ASFV infection, cGAS recognizes viral DNA and synthesizes 2′,3′-cGAMP, triggering the production of interferon to interfere with viral replication. ASFV protein EP364R and C129R have the ability to hinder the host antiviral response triggered by 2′,3′-cGAMP. These proteins not only suppress IFN-I secretion by interacting with 2′,3′-cGAMP but also increase their effectiveness in degrading 2′,3′-cGAMP [101] (Figure 7). At the same time, ASFV EP364R has a homologous domain with the interaction 2′,3′-cGAMP with STING and is able to competitively with 2′,3′-cGAMP [101] (Figure 7). ASFV B175L can interfere with the interaction between cGAMP and STING by interacting with cGAMP, thereby inhibiting downstream signaling of IFN-mediated antiviral response [102].

### 4.3. Targeting STING Inhibits the Molecular Mechanism of cGAS-STING Activation

STING is a 40 kDa adaptor protein located in the endoplasmic reticulum or Golgi apparatus [128, 129]. STING consists of a transmembrane N-terminal domain and a globular C-terminal domain (CTD), and the N-terminal contains four transmembrane helical structures (TM1−4) anchored to the endoplasmic reticulum or other organelles. The C-terminal CTD region is comprised of a ligand-binding section (LBD) and a C-terminal tail segment (CTT). LBD combines 2′,3′-cGAMP and CDNs. CTT can bind TBK1 [130, 131].

**Figure 8:**
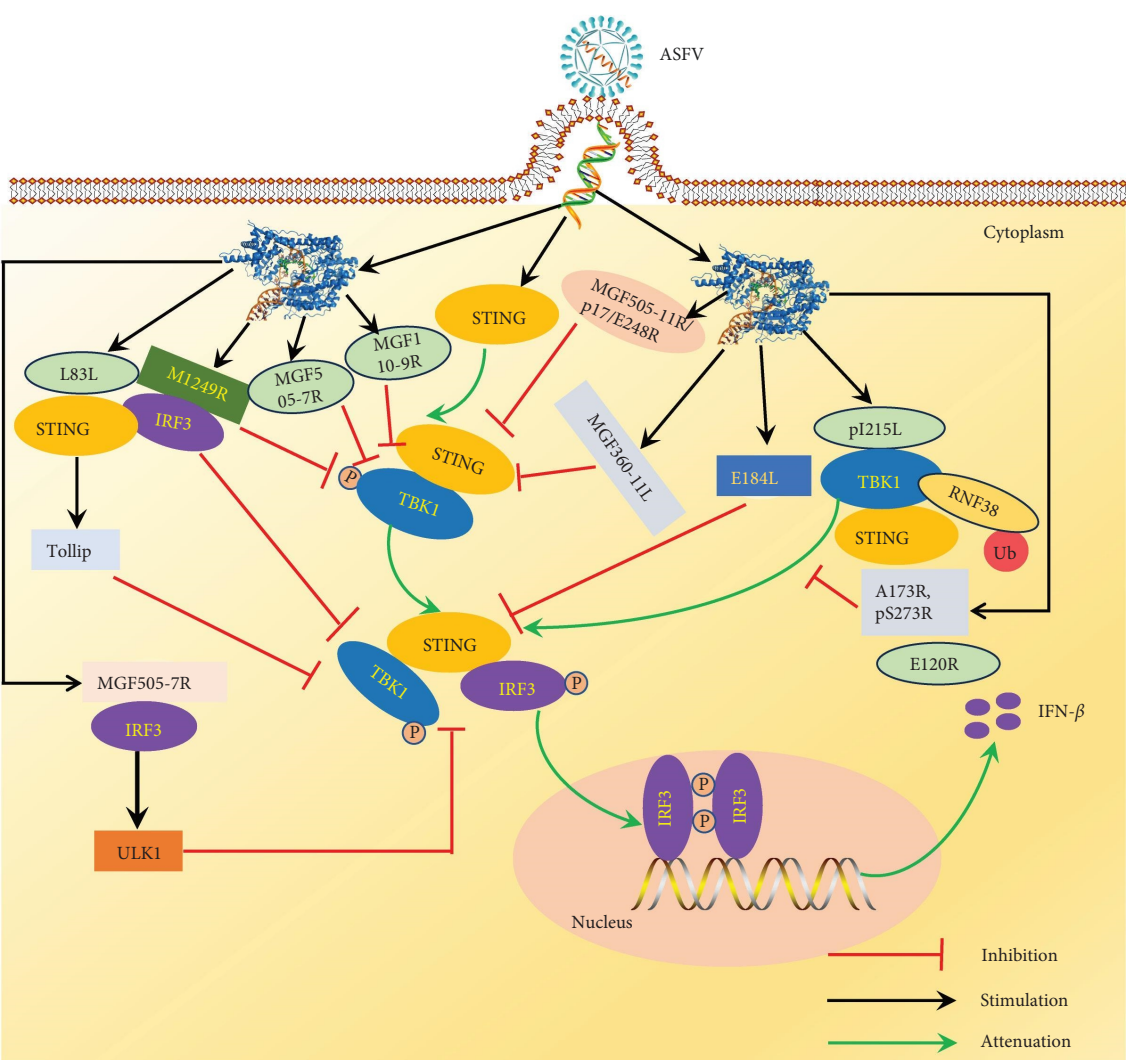
ASFV proteins modulating STING, TBK1, and IRF3 inhibit the molecular mechanism of cGAS-STING signaling pathway activation.

ASFV MGF505-11R suppresses the activation of IFN-*β* and ISRE by cGAS, IRF7, IRF3, STING, IKK-*ε*, TBK1, leading to decreased mRNA transcription of IFN-*β*, ISG15, and ISG56 [106] (Figure 8). MGF505-11R was discovered to have the ability to degrade STING, with the functional domains responsible for this function being 1-191 aa and 182-360 aa of MGF505-11R [106] (Figure 8). E248R binding to STING and blocking the expression of STING suppresses to promote viral replication [105] (Figure 8). ASFV p17 (39-59aa) inhibited the cGAS-STING pathway by interfering with STING recruitment of TBK1 and IKK*ɛ* through its interaction with STING [107] (Figure 8). ASFV L83L overexpression suppresses IFN-*β* promoter and ISRE function, while reducing L83L levels increases ISGs expression and IRF3 phosphorylation in primary porcine alveolar macrophages [103]. L83L enhances the autophagolysosomal breakdown of STING by engaging cGAS and STING to bring in Tollip, inhibiting the phosphorylation of TBK1, IRF3, and IKB-*α*, which are downstream signaling molecules [103] (Figure 8). The ASFV E184L protein suppresses the innate immune responses of the host by interacting with the STING-mediated signaling pathway. E184L hinders the dimerization and oligomerization of STING by interacting with it [108]. Moreover, E184L hinders the assembly of the STING-TBK1-IRF3 complex, leading to the suppression of STING phosphorylation, IRF3 dimerization, and movement into the nucleus [108] (Figure 8). ASFV B175L can interfere with the interaction between cGAMP and STING by specifically interacting with R238 and Y240 amino acids of STING, thereby inhibiting downstream signal transduction of IFN-mediated antiviral response [102]. pH240R binds to the N-terminal transmembrane region of STING, preventing its oligomerization and movement from the endoplasmic reticulum to the Golgi apparatus. This interaction also blocks the phosphorylation of IRF3 and TBK1, leading to reduced production of type I interferon and ultimately promoting viral replication [132].

### 4.4. Targeting TBK1 Inhibits the Molecular Mechanism of cGAS-STING Activation

TBK1 is the center of antiviral innate immune signal transduction. On the one hand, TBK1 can be activated by multiple PRRS. Conversely, TBK1, a crucial enzyme, phosphorylates multiple targets like IRF3 and IRF7 when activated, triggering the start of an antiviral innate immune reaction.

ASFV DP96R blocks cGAS/STING and TBK1, while not affecting IRF3-induced IFN-*β* and ISRE promoter activation [117]. DP96R inhibits TBK1 phosphorylation and inhibits its induced antiviral response [117]. M1249L blocks TBK1 phosphorylation induced by excessive cGAS and STING expression [110]. M1249L can also colocate and interact with IRF3 to induce IRF3 degradation and inhibit host antiviral response through the lysosome pathway [110] (Figure 8). ASFV pI215L is a viral E2 ubiquitination binding enzyme. Reducing pI215L levels suppresses ASFV replication and boosts the production of IFN-*β* [111]. Additionally, pI215L induces polyubiquitination at K63 of TBK1, leading to the inhibition of type I IFN production through its interaction with RING finger protein 138 (RNF138) [111] (Figure 8). MGF360-11L binds with TBK1 and IRF7, leading to the degradation of both TBK1 and IRF7 [118]. Meanwhile, MGF360-11L also inhibits TBK1 and IRF3 phosphorylation to antagonize the molecular mechanism of type I interferon-mediated antiviral activity [118]. MGF505-7R inhibits antiviral activity by interacting with IRF7 and TBK1, degrading IRF7 via autophagy, cysteine, and proteasome pathways, and degrading TBK1 via the proteasome pathway [133] (Figure 8). Deletion of MGF110-9L reduced the virulence of ASFV in pigs and provided complete protection against the attack of parental lethal ASFV [119]. It was found that MGF110-9L/505-7R blocks the degradation of TBK1 through the autophagy pathway and promotes viral replication by targeting the autophagy associated protein PIK3C2B. MGF110-9L promoted the degradation of TBK1 through the autophagy pathway [119] Figure 8). PA151R negatively regulates IFN-I production. Additional research revealed that PA151R and TBK1 engage in a competitive interaction with the E3 enzyme TNF receptor-related protein TRAF6, leading to the suppression of TRAF6 expression via the apoptosis pathway. This ultimately results in the inhibition of TBK1 phosphorylation and the suppression of ASFV replication [113]. PA151R negatively regulates IFN-I production. Additional research revealed that PA151R and TBK1 engage in a competitive interaction with the E3 enzyme TNF receptor-related protein TRAF6, leading to the suppression of TRAF6 expression via the apoptosis pathway. This ultimately results in the inhibition of TBK1 phosphorylation and the suppression of ASFV replication [109].

### 4.5. Targeting IRF3 Inhibits the Molecular Mechanism of cGAS-STING Activation

IRF3 is expressed constitutively in various tissues and cells. When the cells are resting, IRF3 is in the cytoplasm and a self-inhibition state. Following the intrusion of the virus, TBK1 and IKK*ε* phosphorylate numerous serine and threonine sites at the C-terminus of IRF3. After phosphorylation, conformational changes occur in IRF3, and the C-terminal combines to form a dimer, which migrates from the cytoplasm to the nucleus. CBP, a coactivator that binds, stimulates the production of IFN-I.

Another study showed that the molecular mechanism of ASFV DP96R inhibits IRF3-mediated antiviral immune response. DP96R blocks the interaction between activated IRF3 and KPNA, inhibiting the translocation of IRF3 to the nucleus by binding to a crucial site on KPNA within IRF3 [120]. DP96R overexpression enhances DNA and RNA virus replication by inhibiting the cGAS-STING signaling pathway and suppressing antiviral gene transcription. Knocking out DP96R with DP96R-specific siRNA leads to increased IFN and ISG transcription during ASFV infection [120]. ASFV pS273R is a cysteine protease specific to SUMO-1, and it hinders the production of IFN-I by interacting with IRF3 [112], pS273R disrupts the interaction between TBK1 and IRF3, inhibiting the phosphorylation and dimerization of IRF3 [112] (Figure 8). A137-deficient ASFV virus can induce higher IFN-I production [116], and A137R interacts with TBK1 to promote autophagy-mediated TBK1 lysosomal degradation, thereby blocking IRF3 nuclear translocation, resulting in reduced IFN-I production [116] (Figure 8). The ASFV E120R protein is capable of inhibiting the phosphorylation of IRF3 and the production of IFN-I by binding to IRF3 and preventing its recruitment to TBK1 [121] (Figure 8). Elimination of the MGF505-7R gene from ASFV may lead to increased production of IL-1*β* and IFN-*β*. MGF505-7R hinders NF-*κ*B activation by interacting with IKK*α* and suppresses inflammasome formation by binding to NLRP3, leading to decreased IL-1*β* production [134]. MGF505-7R can interact with IRF3, preventing the nuclear translocation of IRF3, and increasing the expression of autophagy-related protein ULK1 to break down STING [104–134] (Figure 8). pD129L inhibits the IFN signaling pathway by disrupting the interaction between transcriptional coactivators p300 and IRF3, leading to the suppression of IFN-I production and enhancement viral replication [124].

## 5. Conclusions and Perspectives

The development of a novel and highly effective ASFV vaccine is the primary problem for researchers around the world. At present, the research progress of live attenuated ASFV vaccine, adenovirus vaccine, recombinant pseudorabies virus vaccine, and antiviral drugs has been made, and the vaccines and drugs have certain protective effects against the attack and replication of ASFV parent strains [90, 135–141]. For example, the attenuated ASFV vaccine HLJ/18-7GD was capable of providing effective immune protection against different wild strains [142]; ASFV genotype II Georgia*Δ*DP148R*Δ*K145R*Δ*EP153R-CD2v mutant Q96R/K108D attenuated strain is able to provide 83%–100% immune protective efficiency against parental strain for different doses of challenge [143]. These results showed that HLJ/18-7GD and Georgia*Δ*DP148R*Δ*K145R*Δ*EP153R-CD2v_mutantQ96R/K108D have potential as candidate vaccines. It is worth noting that, vaccines are needed to provide cross-protection against different ASFV genotypes.

The most striking feature of host defense is that once infected with these viruses, cells can rapidly activate cGAS-STING to produce an innate immune response to ASFV to prevent viral infection. The host defense mechanism is essential for the balance between survival and death of the host. However, the lack of detailed mechanisms of ASFV and host interaction has greatly limited the development of effective vaccines. ASFV proteins inhibit IFN-*β* production by targeting different molecules of the cGAS-STING pathway. Therefore, it maybe impractical to develop drugs that target different viral virulence proteins and apply them to clinical prevention and control. Kuchitsu et al. [144] found that the host VPS protein can promote the transport of STING from the Golgi apparatus to the lysosome, reduce the residence time of STING in the Golgi apparatus, and thus inhibit the immune activation state of the host. In the future, the differences in susceptibility and pathogenicity of ASFV in different animals can also be studied clinically, and the key host proteins that differ in susceptibility and pathogenicity of ASFV can be screened using high-throughput and single-cell sequencing methods, to establish a knowledge base on the host susceptibility system of ASFV.

mRNA vaccines (the third-generation vaccine) have made a great contribution in blocking SARS-CoV-2 infection and transmission [145–147]. mRNA vaccines activate the dual mechanism of humoral and T cell immunity, strong immunogenicity, no need for adjuvants, and ease of mass production support the key advantages of global supply [148, 149]. There are also many studies on mRNA vaccines for DNA viruses, such as the monkeypox virus [150–155] and Epstein–Barr virus [156, 157], which suggests that mRNA vaccines of DNA viruses have a good prospect. ASFV p34, p72, p54, p30, and CD2v are the main immunogenic proteins and can effectively stimulate the body's immune response [143, 158, 159]. Meanwhile, Deletion B125R [160], C84L [161], L11L/L7L [162], DP71L [163], DP96R [163], B119L [164], and DP148R [163] significantly reduced the virulence of the virus and showed partial or complete protection against infection with ASFV strains. Besides, a lot of conserved B cell linear epitope in pB602L [165], p34 [166], p30 [167], pA104R [168], and p72 [169] have been identified. About the ASFV vaccine: (1) we can screen the optimal deletion combination of virulence genes (B125R, C84L, L11L/L7L, DP71L, DP96R, B119L, and DP148R) to obtain attenuated and attenuated vaccines; (2) we can concatenate mRNA vaccines that express different immunogenic proteins (p34, p72, p54, p30, and CD2v); and (3) we can also tandem mRNA vaccines that express different B cell linear epitopes (pB602L, p34, p30, pA104R, and p72) of the same immunogenic protein.

## Figures and Tables

**Table 1:** ASFV's immune evasion tactics on the cGAS-STING signaling pathway.

Target pathway moleculars	Viral proteins	References
cGAS	I226R, QP383R	[99, 100]

2′,3′-cGAMP	EP364R, C129R, B175L	[101, 102]

STING	MGF-505-7R, MGF-505-11R, E248R, p17, L83L, E184L, B175L	[102–108]

TBK1	DP96R, MGF-505-7R, pI215L, MGF360-11L, A137R, S273R, M1249L, MGF360-12L, S273R, MGF-110-9L, pA151R	[109–119]

IRF3	MGF-505-7R, MGF360-14L, I226R, S273R, M1249L, E301R, MGF360-12L, S273R, D129L, E120R, DP96R	[100, 109–124]
